# Clinical records after asynchronous and synchronous e-learning courses: a multi-method randomised controlled trial on students’ performance and experience

**DOI:** 10.1186/s12909-023-04528-2

**Published:** 2023-08-18

**Authors:** Simone Battista, Laura Furri, Giorgia de Biagi, Lucia Depedri, Valeria Broggi, Marco Testa

**Affiliations:** 1 Department of Neurosciences, Rehabilitation, Ophthalmology, Genetics, Maternal and Child Health, University of Genova, Campus of Savona, Savona, Italy; 2 School of Medicine and Surgery, University of Verona, Verona, Italy; 3 School of Medicine and Surgery, University of Milano, Milano, Italy

**Keywords:** Clinical teaching, Healthcare professionals, Rehabilitation, e-learning, Education, distance, Reflective writing, Blended learning, Speech therapy

## Abstract

**Background:**

Clinical Record (CR) writing is a fundamental skill for healthcare professionals, but the best e-learning methods for teaching it remain unstudied. Therefore, we investigated speech therapy students’ differences in the quality production of CR at the placement and their experience after following asynchronous or synchronous e-learning courses.

**Methods:**

A multi-method randomised controlled trial. Fifty speech therapist students were equally and randomly divided into two groups attending asynchronous or synchronous e-learning classes to learn how to write a CR. The quality of the CR was tested through an ad hoc checklist (score 0–32) and the groups’ scores were compared. The assessors and the statistician were blinded to students’ group assignment. Students’ experience was assessed through semi-structured interviews analysed with a reflexive thematic analysis.

**Results:**

No score differences between the two groups were found (Cohen’s d = 0.1; 95% Confidence Interval [-0.6; 0.7]). Four themes were generated: (1) ‘Different Forms of Learning Interaction’, as the synchronous group reported a positive experience with being fed back immediately by the lecturer, whereas the asynchronous group reported that pushing back the question time allows for reflecting more on the learning experience; (2) ‘Different Ways to Manage the Time’, as the synchronous group had to stick to the lecturer’s schedule and the asynchronous group felt the possibility to manage its time; (3) ‘To Be or Not To Be (Present)?’ due to the different experiences of having (or not) the lecturer in front of them; (4) ‘Inspiring Relationships With The Peers’, where both groups preferred a peer-to-peer discussion instead of contacting the lecturer.

**Discussion:**

Asynchronous and synchronous e-learning courses appeared equally effective in teaching CR writing. However, students perceive and experience these methods differently. The choice or blend of these methods should be based on students’ needs and preferences, teacher input, as well as organisational requirements rather than solely on students’ attended performance.

**Supplementary Information:**

The online version contains supplementary material available at 10.1186/s12909-023-04528-2.

## Introduction

Clinical Records (CR) are fundamental to keeping track of patients’ conditions, transmitting essential information among healthcare professionals working on a team and from a legal point of view [1]. Writing them properly is a core skill for future healthcare professionals [2]. Tutors foster the use of CRs during the placement to promote an array of different skills among healthcare students [1, 2] Examples of these skills are applying theoretical concepts from various disciplines in inpatient/outpatient care and interpreting, linking and presenting data that need to be written after actively reflecting upon them [1, 2].

In the last few years, online resources opened up many innovative possibilities for teaching, with distance and blended lessons, whether delivered synchronously or asynchronously, becoming increasingly frequent in healthcare professional degree courses [3, 4]. Asynchronous learning is the use of online platforms that allow students to interact through different online tools such as emails, forums or discussion groups, which, therefore, do not need the simultaneous connection of teachers and learners [5]. Conversely, the synchronous learning mode is characterised by the simultaneous presence of lecturers and students, online [5].

Regarding the difference between these two teaching methods, several studies concluded that the educational and assessment scores reached by students (when it comes to different topic knowledge) were similar for both groups, and there was no evidence that the asynchronous online delivery of the module content disadvantaged one group over the other [5–9]. Moreover, both methods have proven to be an acceptable alternative to face-to-face methods for students and academics [7].

Regarding the production of CRs, there is no evidence of the best e-learning way to teach this skill, whether synchronously or asynchronously. Furthermore, no studies explored students’ experiences of attending CR-related courses online. Thus, this study investigated the differences in the quality of the production of CR among speech therapy students and their experience after following an asynchronous e-learning course compared to a synchronous one through a multi-method randomised controlled trial.

## Materials and methods

### Trial design

A multi-method randomised controlled two-arms parallel-groups trial was conducted, wherein the assessors and the statistician were blinded to the groups the students were assigned. This study is reported in line with the Consolidated Standards of Reporting Trials [10] for the quantitative part and the Consolidated Criteria for Reporting Qualitative Research for the qualitative part [11]. This study was approved by the ethics committee of the Human Sciences Department of the University of Verona (17 February 2021, code 2021_04).

### Setting and participants

This trial was conducted at the Bachelor of Science (BSc) degree course in ‘Speech Therapy’ at the University of Verona (Verona, Italy). The targeted study population consisted of third- and second-year students who attended the second semester of this three-year course before starting the placement. Participants were eligible if they were students enrolled in the third or second year of the abovementioned BSc programme and were willing to participate in the study. Hence, a potential cohort of 50 students was identified: 25 from the second year and 25 of the third year. Information about the study’s objectives was thoroughly provided to the students, after which their willingness to participate was ascertained through written informed consent. In case of unwillingness, they were told that they would receive the synchronous course in preparation for the placement but that their data would not be collected.

### Interventions

Participants were randomised in a 1:1 ratio into one of the two groups described below. The two groups were composed of students in the second and third years. Group 1 attended an asynchronous course, whereas Group 2 attended a synchronous one. Both courses took place in March 2021. Both classes were held by two speech therapists and tutors of the course (GDB and LD). They are both experts in speech therapy clinical practice and were thoroughly trained in both synchronous and asynchronous didactics methods. Regardless of the administration way, the two classes aimed at providing the students of the second and third year of the BSc in ‘Speech therapy’ with the necessary knowledge to produce a CR that contained the assessment and the treatment of a patient they would see during their placement [12, 13]. Students were invited not to share materials or information from the lessons they had to carry out. To improve their compliance, they were informed that, at the end of the experiment, they would carry out the other e-learning course they did not attend during the trial. This decision was also made to allow both groups to receive the same learning opportunities. No changes to the initial protocol submitted to the ethics committee were made.

### Intervention group

The intervention group received an asynchronous course conducted by LD. The asynchronous course was carried out through the use of Google Classroom. The course consisted of four modules with videos that lasted a maximum of 15 min each. The first module presented the course. The second one explained how to write a CR from a formal point of view (paragraphs, writing style, syntactic form etc.). The third and fourth modules dealt with the contents of the CR and the skills students were supposed to achieve by writing it [12, 13]. At the end of each video, there was an assignment followed by feedback from the lecturer. The passage to the successive module occurred only after completing the previous one. In case of need, contacting the lecturer through the platform and email was possible.

### Control group

The control group received a synchronous course conducted by GDB. The synchronous lesson lasted 2 h, was conducted via ‘Zoom’ in March 2021 and consisted of a frontal lecture using slides and a mobile audience response system (i.e., ‘Mentimeter’). The topics covered were the same as those treated in the asynchronous intervention group. The lectures in the intervention (asynchronous) group were shorter than those in the control group because it is recommended to shorten the duration of asynchronous lectures compared to face-to-face ones to enhance the effectiveness of asynchronous teaching [14].

### Randomisation

An external person to this study generated a random allocation sequence using a computer-generated random number list with simple randomisation (www.random.org). The randomisation process was stratified by the year of the study so that the same amount of students from the second and third years was assured in the two groups. The sequence was used to allocate participants to the intervention and control groups. Therefore, the participants eligible for the study were randomly assigned to the two (synchronous and asynchronous) groups (allocation ratio 1:1).

### Outcome

#### Primary outcome – quality of CRs

The primary outcome was the differences between the two groups in the quality of production of a CR that contained the assessment and the treatment of a patient they saw during their placement. A literature search was conducted to look for a checklist to assess CR. Since no checklists were retrieved, an ad hoc checklist was developed following a similar strategy to the one reported by Rossettini et al. [15] by investigating guidelines and speech therapy ethical codes for CRs in the speech therapy field [12, 16–22]. Supplementary Material 1 thoroughly reports how the checklist was created.

The checklist comprised 16 items divided into two parts: a formal and a content one (6 in the formal part and 10 in the content part). Briefly, the former evaluated the presence or absence and the adequacy or inadequacy of some formal characteristics (font, heading, meeting the deadlines, syntactic form, appropriate language etc.) that needed to be present in the CR. The latter evaluated the contents of the CR based on the skills that the students were supposed to reach (patients’ assessment and treatment).

The preliminary version of the checklist was then validated for face and content validity through a Delphi Procedure [23] (see Supplementary Material 1 for a thorough explanation) with eight speech therapists with experience in teaching and the speech therapy clinic. After two rounds, a consensus (> 70%) was reached [24–26]. The panel agreed that all questions would be scored equally with a 3-point Likert scale (0, incorrect; 1, partially correct; 2, correct) and that all items were consistent with the aim of the checklist. Therefore, the total scores of the checklist ranged from 0 (substantially incorrect performance) to 32 (utterly correct performance). The pass score was set at 18 points out of 32. With a sum equal to or greater than 31, honours were attributed.

GDB and LD then tested the final checklist to evaluate the CRs of 10 students not involved in the study. Thus, Cronbach’s alpha was calculated to estimate internal consistency, and the intraclass correlation coefficient (ICC) was calculated to assess Inter-rater agreement. The internal consistency was adequate with Cronbach’s alpha α = 0.82 (95% confidence interval (CI) [0.56–0.94]). Inter-rater agreement was also high (ICC 0.96, 95% CI [0.85–0.99]).

Finally, two blinded assessors used the checklist to evaluate the primary outcome. One had experience in speech therapy clinical practice for developmental age and assessed the 25 reports in that area, and one in speech therapy clinical practice for adulthood and corrected the remaining 25 reports focussed on adults. The evaluators were unaware of the study objectives and participants’ groups and were preliminarily trained using the checklist. The evaluators independently assessed the recorded performance. A comparison between the two groups was made based on the marks obtained. Moreover, upon completion of the data collection processes, feedback on the quality of the report was offered individually to all students in both groups.

#### Secondary outcome – students’ experience

Semi-structured interviews were conducted with each student to investigate students’ experience of attending asynchronous or synchronous lectures. The interviews were performed online, by videoconferencing, and they were conducted only with the interviewee. GDB and LD produced and transcribed an audio-visual recording of each interview verbatim. Questions were asked about the feelings, emotions and impressions that the students have experienced in participating in the synchronous and asynchronous lessons.

The interview guide (Supplementary Material 2) was created by a pool of expert tutors and lecturers, including two speech therapists (GDB and LD) and two physiotherapists and adjunct lecturers at the University of Verona (SB and LF). GDB, LD, and LF identify themselves as women. SB identifies himself as a man. They are all trained in teaching and tutoring. SB is a research fellow and PhD in Neurosciences and Medical Science. He is trained in qualitative research and has provided the other researchers with all the necessary knowledge to perform the interview analysis. The interview guide consisted of open questions exploring different topics related to synchronous and asynchronous teaching modes and students’ experiences.

The semi-structured interviews were performed by GDB and LD and lasted approximately 15 min each. GDB performed the interviews with the students who participated in the asynchronous course, whereas LD performed the interviews with those who participated in the synchronous course. Participants were aware of their professional background and knew GDB and LD as lecturers.

### Data and statistical analysis

#### Primary outcome (quality of CRs)

Statistical analyses were performed by one researcher (SB) that did not know to which group participants belonged. Descriptive analysis was carried out to describe the participants’ profile, evaluating the homogeneity between the groups’ data and the demographic variables of the sample (age, gender they identified with, year of course). Continuous variables were reported as mean ± standard deviation (SD), whilst categorical variables were reported as absolute and percentage frequencies.

Since data did not follow a normal distribution after inspections of q-q plots (stata function ‘qnorm’) between-group analyses were performed using the Mann-Whitney test (stata function ‘ranksum’) to assess differences between the two groups (synchronous and asynchronous) in the quality production of their CRs at the placement. Effect sizes were calculated and reported following Cohen’s d [27, 28]. Moreover, we used bootstrapping (500 replications) to compute effect size’s 95% Confidence interval (CI) (stata formula ‘esize’, reps 500, seed 111) since ‘ranksum’ does not provide the 95% CI directly. The analysis was performed with Stata 17 (StataCorp. 2021. Stata Statistical Software: Release 17. College Station, TX: StataCorp LLC).

#### Secondary outcome (students’ experience)

Data were analysed following the six steps of the ‘Thematic Analysis’ reported by Braun and Clarke (Table 1) [29]. We used thematic analysis as the study aimed at generating patterns in students’ experience of the asynchronous and synchronous courses they attended to write CRs. Thematic analysis is an independent qualitative descriptive approach described as “a method for developing, analysing and interpreting patterns across a qualitative dataset, which involves systematic processes of data coding to develop themes” [29]. Among the various ‘Thematic Analysis’ approaches (i.e., coding reliability, codebook approaches, reflexive approach), we adopted a ‘Reflexive Thematic Analysis’ that is characterised by a coding process that is open and organic, without any coding framework and that emphasises the active role of the researcher in data interpretation and theme generation [29, 30]. RTA posited a ‘Big Q’ qualitative paradigm characterised by adhering to a non-(post)positivist paradigm [31]. Thus, some practices do not apply to RTA (e.g., consensus coding, inter-coder reliability, data saturation, member checking etc.) as they are infused “with assumptions about the nature of reality and meaningful knowledge” that follow a ‘small q’ (postpositivist) paradigm [32, 33].

**Table 1:** Steps of the ‘Reflexive Thematic Analysis’

Phases	Authors’ contribution and action
1) Data familiarisation	All authors read and reread several times the transcriptions of the interviews. This process is fundamental to getting in contact with the data and taking notes of any impressions and insights.
2) Coding	GDB and LD systematically coded the data. They adopted semantic data coding.
3) Generating initial themes	GDB and LD generated initial themes separately, clustering similar codes together.
4) Reviewing and refining themes	All authors reviewed the coding and initial themes separately and then jointly and generated five themes that fit the most data. GDB and LD reviewed the agreed themes against the codes and the entire dataset.
5) Defining and naming themes	All authors finalised the final themes and their definitions.
6) Producing the report	GDB and LD selected illustrative quotations from the interviews, and all authors reviewed and agreed. SB led the writing of the paper, and all authors participated in this phase.

The use of thematic analysis in this study was majorly inductive, as we took the dataset as starting point for our data analysis [29]. We adopted an experiential qualitative framework because we illustrated the characteristics of the students’ experience after attending either an asynchronous or synchronous course on how to write CRs to reflect the perception of social reality (speech therapy students) [29, 34]. We adopted a realist theoretical framework to take the reality as voiced in the dataset [29, 34]. Finally, the data coding was mostly semantic as we stuck to a descriptive level of meaning in the code processing [29, 35]. Multiple strategies were promoted to ensure the rigour and trustworthiness of the data. Firstly, GDB and LD documented field notes (“Memos”) after completing each interview to promote reflexivity [36]. These memos were shared during research meetings for reflexive thoughts. Secondly, the research team met frequently to refine the themes and subthemes the final themes were generated. Thirdly, an audit trail containing meeting notes, analysis discussions, and research decisions was continuously reorganised by the two authors who analysed the interviews (GDB and LD) to stress the dependability and confirmability of the study. Finally, the six steps of the thematic analysis were followed as recommended by Braun&Clarke [29].

### Sample size

#### Primary outcome (quality of CRs)

A priori analysis was run to calculate the sample size needed. It was based on G * Power 3.1 application. Based on other studies on online lecturing, an effect size of d = 0.70 was set [37]. Moreover, the alpha error was set at p = .05 and the power at β = 0.80. Based on a two-tailed unpaired t-test, the total sample size yielded a sample N = 50.

#### Secondary outcome (students’ experience)

All study participants were interviewed, and their interviews were subsequently analysed chronologically based on the dates they were conducted. The analysis was conducted collaboratively by GDB and LD, who maintained continuous communication to refine the coding process and develop subsequent themes. The analysis of interviews was concluded when it was determined that no further interviews were necessary, and the final themes were generated [32]. The choice to stop analysing the interviews aligns with the approach we adopted (RTA). As reported by Braun&Clarke, RTA dwells in uncertainty, acknowledging that meaning is constructed through the interpretation of data rather than simply extracted from it. Therefore, decisions about the quantity of data items and when to conclude data collection and analysis are inherently situated and subjective [38].

## Results

A total of 50 students were included in the study (25 in the intervention group and 25 in the control group). The intervention (asynchronous) group was composed of 24 women (96%) and one man (4%) and 12 (48%) students from the second year, and 13 (52%) from the third year. The control (synchronous) group was composed of 24 women (96%) and one man (4%), 13 (52%) students from the second year and 11 (48%) from the third year). Table 2 reports the sample’s descriptive analysis.

**Table 2:** Descriptive analysis of the whole cohort of students divided per group

	Asynchronous Course	Synchronous Course
**N**	25	25
**Gender (W (%); M (%))**	24 (96%); 1 (4%)	24 (96%); 1(4%)
**Age (Mean ± SD)**	23.48 ± 3.63	23.68 ± 3.10
**Year of the Course (2nd year (%); 3rd year (%))**	12 (48%); 13 (52%)	13 (52%); 12 (48%)

Legend: N, number; W, women; M, men; SD, standard deviation

### Primary outcome (quality of the CRs)

Table 3 reports the score obtained by the two groups. Once comparing asynchronous with synchronous learning modalities, we found an effect size of d = 0.1 [-0.6; 0.7] in the total score, d = 0.3 [-0.3; 0.8] in the formal score and d = 0.2 [-0.5; 0.8] in the content score.

**Table 3:** Students’ score

	Asynchronous Course Median [Q1; Q3]	Synchronous Course Median [Q1; Q3]	Between-Group Analysis	Effect Size (Cohen’s d) [95% CI]	
**Total Score**	30.0 [26.0; 32.0]	30.0 [23.5; 31.5]	U = 289.5 p = .650	d = 0.1 [-0.6; 0.7]	
**Formal Score**	12.0 [11.0; 12.0]	12.0 [11.0; 12.0]	U = 273.5 p = .392	d = 0.3 [-0.3; 0.8]	
**Content Score**	18.0 [15.0; 20.0]	18.0 [13.0; 19.5]	U = 269.0 p = .389	d = 0.2 [-0.5; 0.8]	

Legend: Q1, first quartile; Q3, third quartile

### Secondary outcome (students’ experience)

Among the fifty students who were interviewed, only 30 interviews were analysed (15 from the synchronous group and 15 from the asynchronous group). Descriptive data of this subgroup is reported in Table 4.

**Table 4:** Demographic characteristics of interviewees

Participant	Age	Gender	Course Year	Group
P1	22	W	2	Asynchronous Group
P2	22	W	2	Asynchronous Group
P3	26	W	2	Asynchronous Group
P4	23	W	3	Asynchronous Group
P5	23	W	2	Asynchronous Group
P6	23	W	3	Asynchronous Group
P7	37	W	2	Asynchronous Group
P8	22	W	3	Asynchronous Group
P9	22	W	2	Asynchronous Group
P10	22	W	3	Asynchronous Group
P11	22	W	3	Asynchronous Group
P12	22	W	3	Asynchronous Group
P13	22	W	3	Asynchronous Group
P14	22	W	3	Asynchronous Group
P15	23	W	3	Asynchronous Group
P16	22	W	3	Synchronous Group
P17	31	M	3	Synchronous Group
P18	22	W	2	Synchronous Group
P19	23	W	3	Synchronous Group
P20	23	W	3	Synchronous Group
P21	22	W	3	Synchronous Group
P22	22	W	3	Synchronous Group
P23	22	W	3	Synchronous Group
P24	21	W	2	Synchronous Group
P25	27	W	3	Synchronous Group
P26	22	W	3	Synchronous Group
P27	23	W	3	Synchronous Group
P28	24	W	2	Synchronous Group
P29	33	W	2	Synchronous Group
P30	24	W	2	Synchronous Group

Legend: Async, asynchronous; Sync, synchronous; W, woman; M, man

Analysis of the interview data generated four main themes related to speech therapist students’ experience at the placement following online asynchronous or synchronous courses: (1) ‘Different Forms of Learning Interaction’; (2) ‘Different Ways to Manage the Time’; (3) ‘To Be or Not to Be (Present)?’; (4) ‘Inspiring Relationships With The Peers’.

### Theme 1: different forms of learning interaction

The participants shared the advantages of both asynchronous and synchronous teaching modalities, highlighting how each modality facilitated their learning through distinct mechanisms. Therefore, we generated the theme: ‘Different Forms of Learning Interaction’. Participants in the synchronous group reported a positive experience induced by the possibility of having immediate feedback from the course lecturer. This possibility allowed them to quickly modify, correct, and adjust their assumptions and doubts about the lessons through direct questions.

“It gave us the opportunity to ask questions, to receive live answers, feedback and having someone to talk to. In my opinion, these are important aspects.” (P28, woman, 24y, Synchronous Group).

Instead, the asynchronous group reported that this way of lecturing allows them to reflect more on their learning and knowledge and, therefore, to ask more reasoned questions.

“… whereas in the synchronous lesson, everything happens at the moment, meaning that it becomes a bit more time-consuming if you don’t immediately think of the questions and write them down. In the asynchronous one, on the other hand, you have more time to contemplate the question.“ (Participant 5, female, 23 years old, Asynchronous Group).

### Theme 2: different ways to manage the time

The students reported a common thread in their narration, namely, how they managed their time to deal with their lectures, which differed between the synchronous and asynchronous groups. Hence, we created the theme ‘Different Ways to Manage the Time’. The disadvantage in the synchronous mode was being unable to manage one’s own time and thus being ‘forced’ to be there at that moment. As a result, the students had to stick to the lecturer’s schedule.

“In my opinion, the biggest limitation is perhaps the fact that you are ‘forced’ to be there in that moment. In the sense that if you have an appointment, you clearly don’t have the possibility to see it [the lesson] when you want…” (P23, woman, 22y, Synchronous Group).

Conversely, the students in the asynchronous group positively perceived this modality, primarily due to its flexibility in managing their time based on individual commitments. This flexibility allowed them to engage with the course material at their own pace, accommodating other responsibilities and obligations they may have had.

“Well, certainly, in a period like this, it is much more practical in terms of organisation. I’m not talking about Covid, but the intense period between exams, training, etc. Managing your own time is much more practical.” (P4, woman, 23y, Asynchronous Group).

### Theme 3: to be or not to be (present)?

The students highlighted the impact of the lecturer’s physical presence (or absence) on their learning experiences. Consequently, we generated the theme: ‘To Be or Not To Be (Present)? In the synchronous group, the students experienced the physical presence of the lecturer positively. They felt it was an advantage as having a person in front of them was perceived as stimulating.

“…having a person in front of you is also more stimulating because you are aware of the direct contact, even if it is online. The fact that you know another person is talking to you in real-time, in my opinion, adds something extra compared to just listening to a recording”. (P21, woman, 22y, Synchronous Group)

Moreover, the lecturer’s presence during the synchronous experience allowed the students to feel secure as they felt they had a person to rely on when needed, someone they could interact with in real-time.

“Well, they [the lecturers] are certainly a reference point”. (P19, woman, 22y, Synchronous Group)

In the asynchronous group, the students lacked physical contact with the lecturer. They said that having someone physically in front of them (even if online) makes the experience more “familiar” and less cold and aseptic.

“The disadvantage is that maybe when you have a person in front of you, it’s a little more familiar, let’s say less cold, as an impact…” (P2, woman, 22y, Asynchronous Group).

However, acknowledging this limit, they reported that they did not perceive discomfort in not having the lecturer present during the asynchronous course, conveying a sense of tranquillity.

“I didn’t have any problems. I felt comfortable.” (P7, woman, 37y, Asynchronous Group).

Furthermore, a positive aspect of not having the lecturer physically present was that students felt more at ease, as they were not required to engage in an academic performance that typically involved preparing and being conscious of their physical appearance.

“I am happy with asynchronous because I can stay at home, and I don’t have the social pressure to attend a lesson physically”. (P8, woman, 22y, Asynchronous Group)

### Theme 4: Inspiring relationships with the peers

A shared experience among the students, regardless of their respective groups, was the strong bond they formed with their peers. This bond was so impactful that the students preferred engaging with their peers rather than the lecturer, irrespective of the teaching modalities. As a result, we established the theme: ‘Inspiring Relationship with the Peers’. Moreover, the participants in the synchronous group strongly felt the presence of their peers. The possibility of asking direct questions and relating to peers allowed them to be included in constant confrontations with the other students. So, they felt they could increase the knowledge and skills they learned not only from the lecturer of the course but also from their peers.

“…the beauty of doing a synchronous course is any way this: the interaction, also perhaps with other participants, and also the exchange of ideas, or at least learning from each other’s questions…” (P23, woman, 22y, Synchronous Group).

Irrespective of their group, the interviewees preferred engaging in peer-to-peer discussions rather than contacting the lecturer directly. Consequently, they initially felt more at ease expressing their doubts and concerns to their peers before approaching the lecturer.

“… I did not contact the lecturer directly, but through the course representatives. We brought our doubts and problems in a shared way”. (P19, woman, 23y, Synchronous Group)

## Discussion

This study evaluated the effectiveness of asynchronous and synchronous classes aimed at teaching to speech therapy students how to write a CR during the placement. Moreover, this study explored the students’ experiences of attending those courses. From what was retrieved from this study, it is possible to bring to the forefront that asynchronous lecture is as effective as synchronous one in teaching how to write CRs, as it was already seen for other skills [7, 8] However, differences in the students’ experience were reported in this study.

In the synchronous group, the students asked a question in real time and received immediate feedback from the lecturer. In the asynchronous mode, the answer could only be posed in a second moment through different media outlets (e.g., email, forum etc.) as there is no immediate exchange. However, the possibility of pushing back the question time seemed to allow the students to reflect deeper on what they had learned, making the questions more targeted and well-reasoned. Reflection is a valuable tool that helps students to get the most from their education and other activities [39]. In the synchronous course, students tend to implement the so-called “reflection-in-action” as they do not have the time to process what they have learnt and ask the questions a second time but can only ask the question in real-time [40]. Conversely, the asynchronous course allows for “reflection-on-action” since the learner can process what has been learnt and ask more reasoned questions. A match of these two teaching methods can stimulate both types of reflections in the students.

Another aspect highlighted by the interviews was the different time management between synchronous and asynchronous groups. In the synchronous group, students felt obligated to attend sessions at specific times. In contrast, in the asynchronous group, students appreciated the flexibility to manage their learning based on their commitments. It is worth noting that students’ perceived control over their time was found to correlate significantly with their cumulative grade point average [41]. Macan et al. also pointed out this concept, stressing that the most significant aspect of time management is ‘Perceived Control of Time’ [42]. Their research found that students who perceived that they were in control of their own time reported a significantly more excellent work–life balance, a lower sense of work overload, and less tension than their peers [42]. However, many students struggle to balance their studies and external responsibilities [41]. Thus, perceiving to manage someone’s time might not translate into actual effectiveness in practice.

Then, students in the asynchronous group found this modality much more comfortable, not for being at home per se, but because they did not have to participate in a social performance in front of their lecturer and peers. As a result, the interviewees felt a reduction of social anxiety. Asynchronous computer-mediated communication is often less stressful than real-life interaction because the participants do not need to respond immediately. Social anxiety is lower during online than face-to-face interaction [43]. Experiencing low anxiety levels can facilitate focussed attention and learning in some individuals, particularly when completing routine or relatively simple activities. However, some studies suggest that asynchronous digital communication has momentary benefits for people but that this mode can elicitate anxiety during face-to-face communication among people [44]. Therefore, lecturers should decide which teaching method to adopt based on the skills they want to teach. Suppose it is a technical skill or theoretical knowledge. In that case, they can go for the asynchronous mode because it is not the objective of this lecturer to make the student more confident in managing social anxiety.

As far as the contact with the lecturer is concerned, two different views emerged during the analysis of the interviews. Those who participated in the synchronous group stated that the course tutor’s presence made them feel more guided, safer and less alone. However, despite the lack of contact in the asynchronous group, the students who attended this course reported feeling relaxed in not having anyone in front of them. The students reported that they are now becoming accustomed to this type of teaching and no longer suffer from this type of lesson as they did at the beginning of the pandemic. Therefore, in this case, the blended method can be the best solution to exploit the benefits of both approaches. According to its teaching objectives, the lecturer can select which content to deliver in synchronous and which in asynchronous mode [45].

In our study, interviewees complained about not feeling comfortable enough to express their doubts to the tutors of the course, but they preferred to ask general questions in the group. This attitude can be a disadvantage as there could be a transmission of biassed information and misunderstanding. So, it is the lecturer’s responsibility to find strategies to engage students in the discussion, whether synchronous or asynchronous (i.e., trust, positive and unconditional consideration, respect, acknowledgement, empathy etc.) [45, 46].

The present study has several limitations. First, the participants and the lecturers involved knew to which groups the students belonged. However, the evaluators and the statistician were blinded. Second, our sample comprises speech therapy students; therefore, we are not sure about the transferability/generalisability of our results. Third, the tool used for performance evaluation was explicitly developed by the research team for this study. Despite promising results for the form and content validity processes, further studies are recommended to create a checklist with robust psychometric properties. Fourth, the study was conducted following the signing of an informed consent inviting students not to exchange materials and information between the two groups (synchronous and asynchronous). However, it is not possible to exclude that the students did so. To reduce this possibility, the students were informed that they would change their group at the end of the first placement: the asynchronous intervention group carried out the course synchronously and vice versa. In this way, the students would have obtained all the materials provided by the tutors to each group. The students could revise their CRs after completing the other course. The revised CR was considered for the final grade registered for their university career. Fifth, the students interviewed were in different years of the degree course, and their experience may change during the other years. Finally, standardisation was not possible regarding the CR cases since each student was assigned a different individual during their placement. However, to ensure consistency, we implemented a standardised checklist that focused on evaluating the quality of the reported information rather than the complexity of each analysed case. This approach can be also seen as a strength of the study as it reflects the real-world scenario of a typical placement, where each student is responsible for managing a different patient.

Both synchronous and asynchronous teaching methods have their strengths and weaknesses. Therefore, a blended learning system has been proposed in other teaching areas since it allows students to learn under the best condition [47, 48]. This learning protocol that alternates the two methods offers the students the possibility to enjoy the positive aspects of both teaching ways, reducing the impact of the weaknesses these methods hold. Since, from the results of this study, there are no differences even in teaching how to write CRs properly between synchronous and asynchronous methods, and that both methods have their pros and cons, a possible solution would be to blend these two methods also in this teaching area. When and how using one method instead of the other should be based on the skills the students need to learn, students’ and teachers’ preferences and particular organisational needs rather than students’ performance.

### Electronic supplementary material

Below is the link to the electronic supplementary material.

Supplementary Material 1

Supplementary Material 2

## Data Availability

Data are available upon reasonable request to the corresponding author.

## List of Abbreviations

CR: Clinical record
CI: Confidence interval
RTA: Reflexive thematic analysis
