# Supplementary material for: Clinical records after asynchronous and synchronous e-learning courses: a multi-method randomised controlled trial on students’ performance and experience
Source: BMC Med Educ. 2023 Aug 18;23:584. doi: 10.1186/s12909-023-04528-2 (PMC10439640; doi:10.1186/s12909-023-04528-2)
Supplement: Supplementary file 1 — Supplementary Material 1 [file 12909_2023_4528_MOESM1_ESM.docx]

**Interview Guide**

Good morning,

Thank you for joining us and agreeing to take part in this study. We sincerely appreciate your presence here. As mentioned earlier and outlined in the Informed Consent form, this research and subsequent interview aim to explore any potential disparities in students' experiences regarding clinical records (CRs) production during their placement. Specifically, we aim to examine whether there are differences in CR production experiences when following online asynchronous or synchronous lectures on the topic.

(Please note: the questions asked by LD were posed to the synchronous group; the questions asked by GDB were posed to the asynchronous group).

of

**QUESTIONS**

**GENERAL QUESTION**

1) I would like to start this interview by asking why you chose to do a speech therapy BSc.

1A) In your opinion, what factors influence your study?

**SYNCHRONOUS AND ASYNCHRONOUS MODES**

2A) What was a positive aspect/strength you encountered during your training concerning synchronous and asynchronous teaching methods? And concerning this synchronous (LD)/asynchronous (GDB) specific course for CRs? Did you notice any differences between the previous lessons and the ones we did together?

2B) What was a difficulty/limit that you encountered, during your training, concerning synchronous and asynchronous teaching methods? And concerning this synchronous (LD)/asynchronous (GDB) specific course for CRs? Did you notice any differences between the previous lessons and the ones we did together?

**STUDENT'S EXPERIENCE**

3A) Tell me how you felt not having someone 'face-to-face' during the lessons about CRs. (GDB)

3B) Tell me how you felt having the lecturer present during the lessons about CRs. (LD)

**CONCLUSION**4) Would you like to add an aspect or other elements that came to your mind?
