# Supplementary material for: Clinical records after asynchronous and synchronous e-learning courses: a multi-method randomised controlled trial on students’ performance and experience
Source: BMC Med Educ. 2023 Aug 18;23:584. doi: 10.1186/s12909-023-04528-2 (PMC10439640; doi:10.1186/s12909-023-04528-2)
Supplement: Supplementary file 2 — Supplementary Material 2 [file 12909_2023_4528_MOESM2_ESM.docx]

**The Creation of the Checklist**

To test the primary outcome of this study (quality of the Clinical Record, CR), an ad *hoc* checklist was developed following a similar strategy to the one reported by Rossettini et al. [1] Firstly, two speech therapists (GDB and LD) experts in the speech therapy clinic and two physiotherapists (SB and LF), experts in clinical teaching, searched the literature for references on the knowledge and skills that can be investigated through a CR.[2, 3] In particular, the authors investigated guidelines and logopaedic ethical codes to CRs in the speech therapy field [4–9].

Secondly, they identified elements capable of describing the expected competences that the students needed to reach [4]. The checklist was made up of 16 items divided into two parts: a formal and a content one for a total of 16 items (6 in formal part and 10 in content part). The formal part assessed: the use of a correct layout as asked by the university (e.g., correct heading; Times New Roman font; size: text 12; title size 14; line spacing: 1.5; numbered pages), meeting the deadlines to send out the report, respecting patients’ privacy, the ability to synthesise the document, the clarity and correctness of the syntactic form together with the use of an appropriate clinical language, both specific and scientific, the division of the text into pre-specified paragraphs (personal data, socio-cultural data, environmental and behavioural aspects, significant anamnestic data, clinical diagnosis following icd10 / icd9 codes, instrumental and clinical data, speech therapy evaluation, speech therapy balance, treatment plan, follow up, counselling, conclusions and personal considerations). The content part evaluates the contents of the CR on the basis of the skills that the students were supposed to reach. The characteristics evaluated in this part are the identification of important anamnestic data (demographic, socio-cultural, environmental and behavioural) both in remote and proximal history of the patients; the use of a proper diagnosis following possible international classifications; the quality of the informal speech therapy assessment (i.e., speech therapy interview and observation, both informal and with specific outcome measures); the use of different and adequate assessment tools according to the detected communication, language and swallowing disorders and any specialised investigations and diagnostic investigations; the elaboration of a speech therapy balance relating to the pathologies addressed in which the disorder / diagnosis and the identification of the patient's functional profile are reported; the identification of the rehabilitation goals (both at a short, medium and long term); the identification of the proper treatment plan (counselling, techniques, methods, tools, exercises, verification of results); the identification of adequate identify follow-up times for the assessment and the intervention; an overview and consistency between the various sections: the student presents the clinical case by consequentially linking the paragraphs together, and finally, the clinical reasoning adopted (reflective reasoning is deduced on how the student decided to proceed with the elaboration of the clinical case).

The preliminary version of the checklist was then validated for face- and content-validity through a Delphi Procedure [10] by sending it out to a panel of eight speech therapists, all with experience in teaching and in the speech therapy clinic. GDB and LD sent an email to the panel experts individually, asking them for feedback on the reliability of the checklist. Moreover, they asked if they agreed with the assessment proposal. All experts were unaware of the other participants in the Delphi procedure. In case of doubts, the participants could only email GDB and LD, who would then report the comments received by all the experts to each component individually. After two rounds, a consensus (>70%) was reached (Michels, Evans, & Blok, 2012). The panel agreed that all questions would be scored equally with the Likert scale and that all items were consistent with the aim of the checklist. The panel agreed that all questions would be scored equally with a 3-point Likert scale (0, incorrect; 1, partially correct; 2, correct) and that all items were consistent with the aim of the checklist. Therefore, the total scores of the checklist ranged from 0 (substantially incorrect performance) to 32 (completely correct performance). The pass-score was set at 18 points out of 32. With a sum equal to or greater than 31, honours were attributed.

Thirdly, the final checklist was then tested by GDB and LD to evaluate 10 students not involved in the study. Thus, Cronbach’s alpha was calculated to estimate internal consistency and the intraclass correlation coefficient (ICC) was calculated to assess Inter-rater agreement. The internal consistency was adequate with a Cronbach’s alpha α = 0.82 (95% confidence interval (CI) [0.56–0.94]). Inter-rater agreement was also high (ICC 0.96, 95% CI [0.85–0.99]).

Finally, the checklist was used to evaluate the quality of the CRs as written by students at the placement after following the synchronous and asynchronous lectures, by two blinded assessors. One had experience in speech therapy clinical practice for developmental age and one in speech therapy clinical practice for adulthood. The evaluators were unaware of the study objectives and the participants’ groups. In order to ensure validity, the evaluators were preliminarily trained in the use of the checklist. The evaluators independently assessed the performance recorded. On the basis of the marks obtained, a comparison between the two groups was made. Moreover, upon completion of the data collection processes, feedback on the quality of the report was offered individually to all students in both groups.

**References**

1. Rossettini G, Rondoni A, Palese A, Cecchetto S, Vicentini M, Bettale F, et al. Effective teaching of manual skills to physiotherapy students: a randomised clinical trial. Med Educ. 2017;51:826–38.

2. Mathioudakis A, Rousalova I, Gagnat AA, Saad N, Hardavella G. How to keep good clinical records. Breathe. 2016;12:369.

3. McGeehan R. Best practice in record-keeping. Nursing standard. 2007;21:51–5.

4. Gaudio RM. Autonomia professionale nella valutazione e nella valutazione logopedica. 2010.

5. Mari D. GC, FA, BLK, BG. La scheda di valutazione dell’esperienza di tirocinio: uno strumento di classe. Studio osservazionale nel CLM in Scienze Riabilitative delle Professioni Sanitarie. Journal of Italian Medical Education. 2015;:2942–5.

6. Chiarenza GA, Saccomani L, Cossu G, Loddo S, Penge R, Ruggerini C. Linee guida per i disturbi di apprendimento parte I: I disturbi specifici di apprendimento. www.sinpia.eu. 2011. Accessed 6 May 2022.

7. Holzl J. Twelve tips for effective PowerPoint presentations for the technologically challenged. Med Teach. 1997;19:175–9.

8. Michielin P. Come stendere una relazione diagnostica e di trattamento. Notiziario dell’Ordine degli Psicologi della Regione Veneto. 2007;:20–20.

9. Salerni A, Sposetti P, Szpunar G. La narrazione scritta come elemento di valutazione del tirocinio universitario. Ricerche di Pedagogia e Didattica Journal of Theories and Research in Education. 2013;8:9–26.

10. Michels MEJ, Evans DE, Blok GA. What is a clinical skill? Searching for order in chaos through a modified Delphi process. Med Teach. 2012;34:e573–81.

# Attachments: Checklist

**Clinical Report Checklist**

The following checklist is aimed at evaluating the clinical placement report produced by students of the BSc in Speech Therapy.

It is divided into two parts:

1. FORMAL PART: it evaluates the presence / absence and / or adequacy / inadequacy of some formal characteristics that must be present within a clinical placement report / placement report
2. CONTENTS PART: it evaluates the contents of the clinical placement report / placement report on the basis of the student's skills

The scores range from 0 to 2, where 0 corresponds to "inadequate", 1 corresponds to "partially adequate" and 2 to "adequate". The sum of all the scores obtained gives the mark of the placement report / clinical placement report. The minimum score is 0, while the maximum is 32; with a sum equal to or greater than 31, honors are awarded.

|  | 0 = not adequate | 1 = partially adequate | 2 = adequate | Notes |
| --- | --- | --- | --- | --- |
| **FORMAL PART** | | | | |
| Use of the correct Layout * (see footer) | Respect of 1-2 indicated parameters | Respect of 3-4 indicated parameters | Respect of 5-6 indicated parameters |  |
| Report submission within the indicated deadlines (20 days after the placement has ended) | Sent with a delay of more than 2 days | Sent with a delay of 1-2 days | Sent within the specified time limit |  |
| Respect for privacy | Absent | Partial: respect only for personal or sensitive or highly sensitive data | Present: respect for all patient and caregivers data |  |
| Ability to synthesise | Extremely verbose or concise report (> 10 pages or <2 pages) | Ability to synthesise respected, but superfluous and useless elements are present | Good ability to synthesise |  |
| Clarity and correctness of the syntactic form together with the use of clinical, specific and scientific language | Non-use of clinical, specific and scientific form and vocabulary. | Not always appropriate or partial use of clinical, specific and scientific form and vocabulary. | Clinical, specific and scientific form and vocabulary are used consistently throughout the report |  |
| Text divided into paragraphs as per "Vademecuum" ** (see footer) | Respect of 1-4 of the indicated parameters | of 5-8 of the indicated parameters | Respect of 9-10 of the indicated parameters |  |
|  | 0 = not adequate | 1 = partially adequate | 2 = adequate | Notes |
| **CONTENT PART** | | | | |
| Identify important anamnestic data (remote and proximate anamnesis), personal, socio-cultural, environmental and behavioral aspects of relevance | Insufficient, incorrect or irrelevant data | Data present, but incomplete or not fully clear | Data correct, clear, exhaustive and relevant |  |
| Disorder or clinical diagnosis and possible international classification | Absent or incorrect (e.g., wrong ICD9-ICD10) | Present, but without international classification or without reason | Present, exhaustive and / or with international classification |  |
| Informal speech therapy assessment: speech therapy interview and observation (non-formal / testistic) | Absence of informal - contextual evaluation | Presence of fundamental elements of informal-contextual evaluation that do not allow a complete view of the clinical picture | Presence of the fundamental elements of informal-contextual evaluation: the clinical picture is complete |  |
| Choose the different and adequate assessment tools according to the detected communication, language and swallowing disorders and any specialized investigations and diagnostic investigations | Inadequate and unjustified choice of valuation tools | Partially adequate and partially justified choice of valuation tools | Adequate and exhaustively motivated choice of valuation tools |  |
| Elaborate a speech therapy balance relating to the pathologies addressed in which the disorder / diagnosis and the identification of the patient's functional profile are reported. | Total lack or minimal interpretation of collected data, absence of diagnosis / disorder and functional profile | Discrete interpretation and integration of collected data, absence of diagnosis / disorder or of functional profile | Complete clear and correct interpretation, integration of collected data and presence of diagnosis / disorder and functional profile |  |
| Identify speech therapy rehabilitation goals (short, medium and long term) | Lack of all fundamental objectives for the patient's rehabilitation; objectives that do not conform to the patient's pathology; objective not achievable within the foreseen timeframe; lack of hierarchy in the drafting of objectives | Lack of some fundamental objectives for the patient's rehabilitation; objectives partially consistent with the patient's pathology; timing of goals not always truthful; hierarchy in the drafting of the objectives not always respected | Presence of all the fundamental objectives for the rehabilitation of the patient; objectives in accordance with the patient's pathology; truthful goal timing; hierarchy in the drafting of objectives respected |  |
| Identify the speech therapy rehabilitation intervention / treatment plan (counseling, techniques, methods, tools, exercises, verification of results) | Absence of speech therapy actions (counseling, techniques, methods, tools, exercises, verification of results) or incorrect complent with reference to the patient's pathology | With reference to the objectives identified, the treatment plan is partial: presence of speech therapy actions contingent on the patient's pathology, but incomplete. | With reference to the objectives identified, the treatment plan is characterized by speech therapy actions contingent on the patient's pathology and reported in a complete manner |  |
| Identify follow-up times | Absent or completely incorrect in reference to the patient's pathology | Present, but generic or incomplete by measure of evaluation or timing | Present and complete by measure of evaluation and timing |  |
| Overview / consistency between the various sections: the student presents the clinical case by consequently linking the paragraphs together | Lack of consistency between the various sections | Fair consistency between the various sections | Excellent consistency between the various sections |  |
| Clinical reasoning: reflective reasoning is deduced on how the student has decided to proceed with the elaboration of the clinical case | Completely absent compared to the clinical case | Present, but excessively generic | Present specific and exhaustive (considers all the variables of the specific case) |  |

**Minimum score:** 0

**Maximum score:** 32

**Praise:** > 30

*

Correct header; Times New Roman font; Size: text 12; Title size 14; Line spacing: 1.5; Numbered pages

**

- Personal data, socio-cultural data, environmental and behavioral aspects
- Relevant anamnestic data
- Clinical diagnosis and ICD10 / ICD9 codes
- Instrumental and clinical data
- Speech therapy evaluation
- Logopedic budget
- Treatment plan
- Follow up
- Counseling
- Conclusions and personal considerations
